# Association between Advanced Glycation End Products, Soluble RAGE Receptor, and Endothelium Dysfunction, Evaluated by Circulating Endothelial Cells and Endothelial Progenitor Cells in Patients with Mild and Resistant Hypertension

**DOI:** 10.3390/ijms20163942

**Published:** 2019-08-13

**Authors:** Bogna Gryszczyńska, Magdalena Budzyń, Beata Begier-Krasińska, Angelika Osińska, Maciej Boruczkowski, Mariusz Kaczmarek, Alicja Bukowska, Maria Iskra, Magdalena Paulina Kasprzak

**Affiliations:** 1 Department of General Chemistry, Chair of Chemistry and Clinical Biochemistry, Poznan University of Medical Sciences, 60-806 Poznan, Poland; 2 Department of Hypertension, Angiology, and Internal Disease, Poznan University of Medical Sciences, 61-848 Poznan, Poland; 3 Department of Clinical Immunology, Poznan University of Medical Sciences, 60-806 Poznan, Poland; 4 Medical Analysis Laboratory Regional Blood Centre, 60-354 Poznan, Poland

**Keywords:** oxidative stress, oxidative modification of proteins, receptor sRAGE, endothelium dysfunction, mild hypertension, resistant hypertension

## Abstract

The aim of the present study was to evaluate advanced glycation end products (AGEs) and soluble form of receptor RAGE (sRAGE) concentrations as well as the AGEs/sRAGE ratio in mild (MH) and resistant (RH) hypertensive patients in comparison with normotensive individuals. We also evaluated the association between AGEs, sRAGE as well as AGEs/sRAGE ratio and circulating endothelial cells (CECs) and circulating endothelial progenitor cells (CEPCs). The MH group consisted of 30 patients, whereas 30 patients were classified for the RH group. The control group (C) included 25 normotensive volunteers. AGEs and sRAGE were measured using enzyme-linked-immunosorbent assay (ELISA). The multicolor flow cytometry was used for analysis of CECs and CEPCs. Significantly higher levels of AGEs in RH cohort were observed as compared to C cohort. Furthermore, significantly lower sRAGE levels as well as a higher AGEs/sRAGE ratio were observed between MH and RH cohorts. Significant correlations were found in the MH cohort for sRAGE and CECs, and CEPCs. The elevation of AGEs levels suggests that oxidative modification of proteins occurs in hypertension pathogenesis. The decrease in sRAGE levels and elevation of the AGEs/sRAGE ratio in MH and RH groups may suggest that hypertensive patients are less protected against the side effects of AGEs as a consequence of an insufficient competitive role of sRAGE against the AGEs-RAGE axis. Finally, it may be concluded that the level of AGEs may be an independent predictor of the condition and function of the endothelium. Furthermore, sRAGE may be classified as a potential biomarker of inflammation and endothelium dysfunction.

## 1. Introduction

Hypertension is one of the most complex and chronic diseases in the world. Additionally, patients with hypertension have a significant risk of developing cardiovascular and renal diseases [1,2]. Stages of hypertension have been identified and are applied in clinical diagnosis [3,4,5]. Mild hypertension (MH), also known as grade 1 hypertension, is defined as systolic blood pressure between 140–159 mmHg and diastolic blood pressure between 90–99 mmHg [6]. In contrast, resistant hypertension (RH) is defined as blood pressure measurements that remain above 140/90 mmHg despite use of varying classes of antihypertensive medications, including a diuretic. Resistant hypertensive patients have a higher risk of end-organ consequences, including heart failure, stroke, ischemic heart disease, and renal failure [3,4,6].

The pathogenesis of hypertension is multifactorial and highly complex. Recent work indicates that oxidative stress may be a key participant in the pathogenesis of hypertension [7,8]. The oxidative modification of proteins is an irreversible process which can lead to development of pathological conditions of the vascular system. The non-enzymatic, advanced glycation process of proteins and lipids leads to the formation and accumulation of advanced glycation end products (AGEs) [9,10]. Various adverse effects of AGEs on endothelium function have been demonstrated [10,11]. It has been indicated that AGEs affect left ventricular (LV) remodeling, directly or indirectly, through the cross-linking of collagen and elastin. Further, AGEs may impact LV remodeling via the interaction with cardiac receptors, which in turn lead to increased inflammation and oxidative stress [11]. It was also hypothesized that AGEs may quench nitric oxide (NO), therefore decreasing the bioavailability of NO and resulting in endothelial dysfunction [12]. Finally, the activation of full-length RAGE, a multiligand cell bound receptor, by AGEs increases endothelial permeability and results in procoagulant endothelium [13,14]. Furthermore, the RAGE-ligand complex triggers an inflammatory cascade which promotes the liberation of cytokines, generation of reactive oxygen species (ROS), and synthesis of proteases by the arterial wall [13,14]. In contrast, the function of a “decoy/scavenger receptor,” for full length RAGE is performed by soluble RAGE (sRAGE) by binding pro-inflammatory ligands.

It has been demonstrated that the simultaneous analysis of AGEs and sRAGE is more useful for studying the potential function of the AGE-RAGE axis in diabetes and cardiovascular disease [15,16]. Increasingly, researchers have emphasized the involvement of the AGEs-RAGE axis in human endothelial dysfunction associated with various diseases. Furthermore, it has been reported that the ratio of AGEs/sRAGE may be classified as an independent predictor of endothelial dysfunction, evaluated by flow-mediated vasodilation (FMD) [16]. Despite increasing interest in this field of research, the AGEs-RAGE axis and usefulness of AGEs/sRAGE ratio as prognosticator for elevating endothelial dysfunction remains poorly understood in hypertensive patients. This is particularly evident when assessing disease severity in comparison with the examination of normotensive individuals.

In view of this, the aim of the present study was to evaluate the concentrations of AGEs and sRAGE as well as the AGEs/sRAGE ratio in MH and RH patients as compared to normotensive individuals. We aimed to evaluate the usefulness of the AGEs/sRAGE ratio as a biomarker reflecting AGE-RAGE axis in hypertensive patients. Additionally, we evaluated the association between AGEs & sRAGE as well as AGEs/sRAGE ratio and circulating endothelial cells (CECs) and circulating endothelial progenitor cells (CEPCs) as novel and highly specific markers reflecting the endothelial status. Finally, we identified three potential independent biomarkers of endothelial dysfunction in hypertensive: AGEs, sRAGE and AGEs/sRAGE.

## 2. Results

**Figure 1:**
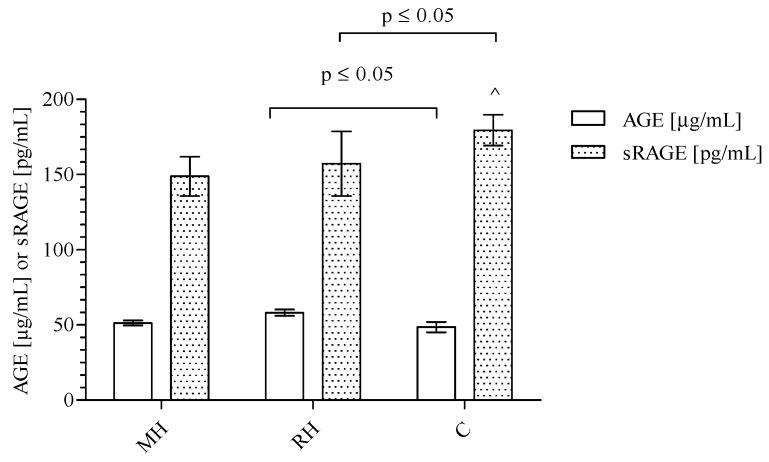
The level of AGEs and sRAGE in the blood of MH, RH, and C groups. The results are presented as mean and standard deviation. The AGEs and sRAGE levels were compared using the Kruskal-Wallis test followed by Dunn’s multiple comparison test. *p* ≤ 0.05 was considered statistically significant.

**Figure 2:**
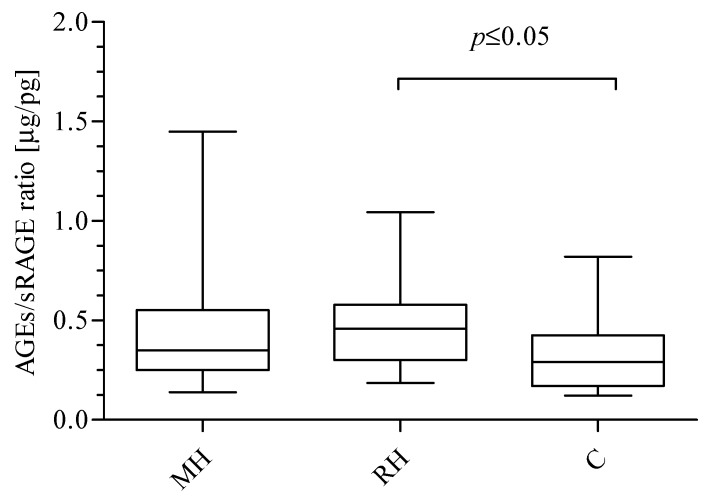
The AGEs/sRAGE ratios in the blood of MH, RH, and C groups. Box and whisker plots show median (central line), upper and lower quartiles (box) and range excluding outliers (whiskers). Data were analyzed using the Kruskal-Wallis test followed by the Dunn’s multiple comparison test. *p* ≤ 0.05 was considered statistically significant.

Among studied groups, significant differences were observed for AGEs and sRAGE concentrations. The data presented in Figure 1 show significantly higher levels of AGEs in RH group as compared to C patients (Kruskal-Wallis test: *p* = 0.0229; Dunn’s Multiple Comparison Test: RH vs. C *p* ≤ 0.05). However, concentrations did not differ significantly in comparison to the MH group. The mean concentration of AGEs was 51.32 ± 9.00 μg/mL (median: 50.70 range: 45.30–57.20) in the MH group, 58.12 ± 11.17 μg/mL (median: 57.00; range: 50.86–63.42) in RH group, and 48.52 ± 14.73 μg/mL (median: 50.30 range: 36.50–56.43) in the C group. Furthermore, the data presented in Figure 1 demonstrate significantly lower sRAGE level in the RH group compared to the C group (Kruskal-Wallis test: *p* = 0.0414; Dunn’s Multiple Comparison Test: RH vs. C *p* ≤ 0.05). No difference in sRAGE concentration between the MH and RH groups was observed, however, there was an observed tendency to have increased concentrations of sRAGE as follows: MH (148.9 ± 70.30 pg/mL; median 144.2; range: 93.43–190.0) < RH (157.2 ± 113.7 pg/mL; median: 123.3; range: 94.50–180.3) < C (179.5 ± 42.39 pg/mL; median: 177.3; range: 146.2–217.7) was found. Next, the ratio of AGEs to sRAGE was calculated (Figure 2). A significantly higher AGEs/sRAGE ratio was found for the RH group (0.48 ± 0.23 μg/pg; median: 0.46; ratio: 0.30–0.59) in comparison with C (0.33 ± 0.18 μg/pg; median: 0.29; range: 0.17–0.43). The trend of a decreasing AGEs/sRAGE ratio was observed, starting with RH patients, followed by MH (0.45 ± 0.30 μg/pg; median: 0.35; range 0.25–0.55) group and ending up with C individuals (Kruskal-Wallis test: *p* = 0.0447; Dunn’s Multiple Comparison Test: RH vs. C *p* ≤ 0.05).

AGEs, sRAGE, and AGEs/sRAGE were analyzed as categorical variables. MH, RH and C patients were divided into three subgroups using 25th and 75th percentiles (group I < 25th percentile, group II and III 25th–75th percentile and group IV >75th percentile) based on AGEs, sRAGE and AGEs/sRAGE levels. The effect of categorization of studied groups on CEC number, CEPC number, SBP1, and hsCRP was analyzed. The data presented in Table 1 show significant differences between quartiles. The insignificant differences are not included. As shown in Table 1, the number of CECs and CEPCs of MH patients decreased across the increasing quartiles of AGEs/sRAGE ratio. Moreover, sRAGE concentration decreased significantly across the increasing quartiles of AGEs in C group.

Significant correlations were found in MH group for sRAGE and CECs, CEPCs, as well as SBP1. Furthermore, in the MH group, the AGEs/sRAGE ratio was found to be significantly correlated with CECs and CEPCs. In the RH group, number of CECs was found significantly correlated with hsCRP and CEPCs. Moreover, a significant negative correlation was found in C group for AGEs and sRAGE. All correlation coefficients in the analyzed groups are listed in Table 2.

In our study hsCRP was used as the criterion to divide MH and RH patients into appropriate groups with low (hsCRP < 1 mg/L), medium (1 mg/L ≤ hsCRP < 3 mg/L), and high (hsCRP > 3 mg/L) inflammatory conditions. The classification of MH patients in to appropriate subgroups, according to hsCRP value, did not differ significantly (Table 3). It was demonstrated, that hsCRP negatively correlated with sRAGE and positively correlated with the AGEs/sRAGE ratio in MH patients with 1 mg/L ≤ hsCRP < 3 mg/L. In RH group, significantly higher AGEs concentration for patients with a high hsCRP level compared to subgroup with 1 mg/L ≤ hsCRP < 3 mg/L was found. Furthermore, a significantly lower sRAGE concentration for RH patients with the highest hsCRP versus the subgroup with low hsCRP was identified. Moreover, a tendency for an increased AGEs/sRAGE ratio with increased hsCRP value in RH patients was also observed. As shown in Table 3, in RH patients with low, medium, and high hsCRP levels had significantly higher SBP1 values than patients in the corresponding MH subgroups. Moreover, significantly lower SBP1 level for RH patients with the highest hsCRP compared to patients with low hsCRP was found.

In the next step, the influence of gender on AGEs, sRAGE, the AGEs/sRAGE ratio, hsCRP and SBP1 in hypertensive patients was estimated (Table 4). In both MH male and MH female patients, all analyzed parameters did not differ significantly. It was, however, demonstrated that the gender of patients affects hsCRP in the RH cohort. As shown in Table 4, in RH males the plasma level of hsCRP was significantly higher than in RH females. It was also shown that the hsCRP value in RH male group was significantly higher than in MH male. A significantly higher AGEs level was demonstrated in RH male patients versus MH male patients. The same relationship for AGEs in RH female patients compared to MH female patients was observed. Moreover, the value of SBP1 was significantly higher in the RH male group versus the MH male group as well as in the RH female and MH female groups.

The influence of AGE on AGEs, sRAGE, the AGEs/sRAGE ratio, hsCRP and SBP1 in hypertensive patients was also analyzed (Table 5). In our study median of age was used as the criterion to divide MH (age = 56) and RH (age = 60) patients into appropriate groups. In both MH ≤ 56 and MH > 56 as well as RH ≤ 60 and RH > 60 all analyzed parameters did not differ significantly.

Finally, two hypertensive groups were combined for the purpose of multiple linear regression analysis in order to provide greater statistical power and to estimate the effect of independent variables on endothelium dysfunction. To this end, five different models were constructed: an unadjusted model (model 1), adjusted for hypertension type (model 2), adjusted for SBP1 (model 3), adjusted for hsCRP (model 4), and adjusted for Cr (model 5). Independently of hsCRP, AGEs level was negatively associated with CECs level, whereas sRAGE concentration with CEPCs/CECs ratio, respectively. All coefficients and *p* values are listed in Table 6.

## 3. Discussion

The oxidative modification of proteins is one of the most adverse processes resulting from oxidative stress which leads to pathological conditions of the vascular system [17]. AGEs, a subclass of oxidatively modified proteins, are postulated not only as a novel marker of oxidative damage but also as a promising predictor of endothelial dysfunction [18,19]. Several lines of evidence suggest that AGEs may play a role in arterial stiffening and hypertension [18,20]. A formation of crosslinks through the collagen molecule may promote a loss of elasticity in the vessel wall [18,21,22].

Interestingly, no significant difference in AGEs levels between MH and RH patients in our study was observed. However, we demonstrated a significantly higher AGEs level in the RH group in comparison to the C group. Multiple explanations concerning the lack of the gradation in AGEs according to the disease severity are plausible. The first assumes that oxidative stress associated with patients in the MH group is more intense, despite significantly lower SBP1 and fewer complications than is characteristic of RH patients. In the previous study, we demonstrated that the oxidative stress plays a crucial role in the pathogenesis of chronic venous insufficiency and, importantly, occurs in early stage of disease development [23]. This interesting concept indicates a possible adaptive mechanism by which inhibition of further oxidation may have taken place in RH patients. It is possible that MH patients are less protected against oxidative stress but this theory would require additional experiments.

It is also worth noting that the aim of our study was to evaluate the concentration of only one of many products of oxidative modification of proteins while, in fact, various types of oxidation reactions may take place simultaneously [24]. It is possible that hypertension promotes different types of protein modifications. Also, it may be argued that the lack of significant differences in AGEs levels between studied groups may be the result of pharmacological agents which may attenuate AGEs formation, prevent AGEs formation or break crosslinks between AGEs and collagen [25,26,27]. Finally, the insignificant differences in AGEs levels between studied groups may point to the endogenous as well as exogenous source of AGEs in human body such as aging, diet, tissue catabolism, renal catabolism and endogenous formation [28,29]. It may be also assumed that a higher number of participants could possibly reveal the effect of the grade of hypertension on the AGEs level. However, further studies would be required to verify any of above-mentioned explanations.

The content of AGEs found in the studied patient groups reflects the rate of formation as well as the rate of removal [29]. The harmful effect of AGEs results from their biological properties and is also related to their ability to interact with specialized receptors, such as RAGE [30]. In contrast, sRAGE has the ability to limit the side effects of RAGE activation. In the present study, the concentration of sRAGE was elevated in healthy volunteers compared to MH and RH patients. Our results are in accordance with observations of Prasad, whose research showed that the plasma level of AGEs is elevated, while the levels of sRAGE are decreased in patients with hypertension [18]. As in the case of AGEs, the lack of difference in sRAGE level between MH and RH patients may suggest that MH patients are less protected against side effects of AGEs, again this theory would require additional experiments.

In the present study, insignificant differences in AGEs/sRAGE ratio between hypertensive patients were observed. However, a trend of decreasing AGEs/sRAGE ratio was observed, starting with RH patients, followed by MH group and ending up with C individuals. Furthermore, a significantly higher AGEs/sRAGE ratio in RH group compared to the C group was found. The lower value of sRAGE per AGEs may suggest an insufficient generation of sRAGE, thus facilitating the binding of ligands, including AGEs, with RAGE receptors.

We also found a negative correlation between AGEs and sRAGE in the C group. Moreover, the categorization of healthy volunteers into quartiles according to AGEs level revealed their effect on sRAGE concentration. The literature data show that serum levels of AGEs correlate positively rather than inversely with sRAGE [30,31]. Nevertheless, these correlations were elevated in pathological conditions, not in case of healthy subjects. It is possible that our results reflect a normal relation between AGEs and sRAGE in healthy conditions. The increase of AGEs concentration and a corresponding decrease of sRAGE concentration indicates the ability of the latter to bind competitively with AGEs in normotensive subjects. Furthermore, the above-mentioned relation confirms the competitive role of sRAGE against AGE-RAGE axis [32]. The lack of differences in AGEs concentration, as well as correlation between AGEs and sRAGE in hypertensive patients suggest that the protective role of sRAGE against hypertensive complications is insufficient.

Some studies indicate associations between serum levels of AGEs, sRAGE, AGEs/sRAGE, and vascular function. Kajikawa and co-authors hypothesized that the ratio of AGEs/sRAGE may be classified as an independent predictor of endothelial dysfunction, evaluated by FMD [16]. Further, Geroldi at al. noted that sRAGE may play a role in arterial stiffening a crucial complication associated with hypertension in patients [25]. In our previous study, we demonstrated an increased CEC numbers and a decreased CEPC/CEC ratio in both MH and RH patients in comparison with normotensive volunteers [23]. Furthermore, a tendency of CEPC numbers to rise in both types of hypertensive patients was observed. In the present study, a significant and positive correlation was found between sRAGE and number of CEPCs in the MH group, which led us to hypothesize that an intensified formation of sRAGE as well as increased numbers of CEPCs confirms the protective mechanisms against progressive endothelium damage. However, in our previous study, we also noticed that the process of endothelial regeneration is inadequately reflected by a significantly lower CEPC/CEC ratio in both MH and RH patients versus C group [23]. We demonstrated that the AGEs/sRAGE ratio is negatively correlated with the number of CEPCs in the MH group. Furthermore, more detailed statistical analysis concerning the categorization of MH patients into quartiles revealed an inverse effect of the AGEs/sRAGE ratio on CEPCs levels.

In agreement with the most recent observation, Chen and co-authors reported that AGEs caused the formation of ROS in CEPCs in dose-dependent manner [33]. They demonstrated that treatment of cultured CEPCs with AGEs showed an increase in NADPH activity, activation of the c-Jun *n*-terminal kinase pathway, which then may promote apoptosis and inhibit CEPCs proliferation. Based on the above-mentioned signaling axis, we hypothesize that the low AGEs/sRAGE ratio corresponding to high CEPCs levels may suggest a sufficient generation of sRAGE, hence the binding of AGEs with RAGE receptors becomes more difficult leading to a decrease of oxidative stress. On the other hand, increasing AGEs/sRAGE may reflect an insufficient competitive role of sRAGE against AGE-RAGE axis, induction of oxidative stress, and consequently endothelial dysfunction reflected by the lowest CEPC numbers.

Oxidative stress and endothelial dysfunction are main factors involved in hypertension. Human and animal studies have confirmed that inflammation leads to development of this disease [34]. The acute phase protein, C-reactive protein (CRP), is an inflammatory marker strongly associated with hypertension [34]. The elevation of hsCRP in hypertensive subjects, as well as in normotensive subjects who are predisposed to hypertension, were found [35,36]. Based on literature data, it is not surprising, that MH and RH patients differ significantly in hsCRP levels. Some researchers have focused on the relationship between AGEs, circulating RAGE levels and hsCRP in different diseases. Interestingly, some authors postulated that low levels of sRAGE may promote the AGEs—RAGE interaction resulting in production of cytokines and, consequently, the increased serum level of CRP [37,38]. In the present study, this activation may be reflected by the positive correlation between CECs and hsCRP levels, which therefore suggests that systemic inflammation plays a significant role on endothelial damage in hypertensive patients. McNair and co-authors demonstrated lower sRAGE levels, while hsCRP and TNF-α concentrations were higher in patients with non-ST-segment elevation myocardial infraction compared to control group [37]. Hamuaty and co-workers found that the protective function of sRAGE reflected an inverse relationship between hsCRP and sRAGE in the early stages of diabetes [38]. In the present study, for the first time to our knowledge, we have demonstrated a significant and reversible association between sRAGE concentrations, AGEs/sRAGE ratio, and hsCRP concentrations in MH patients with medium level of hsCRP (1 mg/L ≤ hsCRP < 3 mg/L). Interestingly, the significant alteration was only observed when groups were sub-categorized. With this sub-categorization assessment of MH revealed a likely relationship with hsCRP, which may reflect the effect of inflammation status on sRAGE level. The insignificant rise in sRAGE levels found for MH with medium hsCRP level versus MH with low hsCRP levels (hsCRP < 1 mg/L) may reflect the increased expression of RAGE on the cell surface. Increased expression of RAGE is known to be a protective mechanism against increasing ligands levels, such as AGEs. Chen and co-authors observed that expression of RAGE increased during exposition of CEPCs on CRP in dose-dependent manner [33]. Similarly, Du Yan and co-authors mentioned that CRP activates nuclear factor kappa B (NF-κB), resulting in increased RAGE expression [39]. In fact, our results appear to agree with both hypotheses mentioned above and suggest that sRAGE levels reflects the inflammation cascade initialized by CRP, resulting in a rise in RAGE expression. We postulate that sRAGE may be a potential factor that reflects the complexity and multifactor character of the pathogenesis of hypertension. Furthermore, increased AGEs levels as well as an increased AGEs/sRAGE ratio and the simultaneous decrease in sRAGE levels might to contribute to accelerated hypertension.

We did not find a correlation between hsCRP and sRAGE in RH patients when they were categorized into appropriate subgroups according to hsCRP value. However, the tendency to exhibit decreased sRAGE concentrations and an increased AGEs/sRAGE ratio with increasing hsCRP in RH patients was observed. It is possible that a larger number of participants would enhance the statistical significance of hsCRP and sRAGE relationship, yet further examination is essential. These data suggest that the association between sRAGE and complications through inflammatory pathway, such as tissue damage which is exacerbated in RH patients, cannot be excluded. On the other hand, the lack of above-mentioned correlation in RH group may be a precursory evidence that sRAGE is not the only modulator of hsCRP in the advanced stage of this disease.

Different risk factors, such as demographics (sex, age and residence), behavioral, and biological factors have been identified for hypertension [40,41,42]. In our study gender was used as the criterion to divide MH and RH patients into appropriate groups, in which AGEs, sRAGE, AGEs/sRAGE ratio, hsCRP, and SBP1 were evaluated. Our study demonstrates a higher concentration of AGEs in RH women as well as RH men versus corresponding MH cohorts, which shows a significant trend to increase according to the disease severity. However, the differences in sRAGE levels and AGEs/sRAGE ratios were not observed. Interestingly, our results show the more intensive inflammation in RH men manifested by an increased plasma hsCRP. It is possible that the inflammation is less pronounced in women than in men due to gradation of disease severity. It has been postulated that blood pressure is higher than in women at similar age [43,44]. Although the mechanisms responsible for the control of blood pressure in men and women are still unclear and differ significantly, there is evidence suggesting that hormones play a crucial role in blood pressure regulation [43]. The lack of female hormones may be the factor contributing to increase in blood pressure in post-menopausal women [43]. In the present study, the age of female participants suggests that rather more women are peri-menopausal or after menopause than pre-menopausal. Although, information on menopause, use of menopausal hormone therapy were not collected, the significant difference in SBP1 between MH and RH women may suggest that an effect of hormonal changes / hormonal imbalances on elevation of blood pressure.

In our study age was used as the criterion to divide MH and RH patients into appropriate groups, in which AGEs, sRAGE, the AGEs/sRAGE ratio, hsCRP, and SBP1 were evaluated. Interestingly, the influence of age on analyzed parameters was not observed. Some studies indicate an association between the AGEs level and the development of chronic degenerative disease of aging, such as Alzheimer’s disease [45,46]. On the other hand, some authors postulated that the reduction of intake and the AGEs level in the blood may promote healthy aging and greater longevity [47,48]. It should also be emphasized that the level of AGEs is defined by the rate of the formation and the rate of elimination [28]. Based on the literature data, it may be concluded that AGEs form during aging, and in hyperglycemic patients contribute in pathophysiology of vascular system [49]. The formation of AGE cross-linking of collagen and elastin results in loss of elasticity in the vessel wall, intensify of oxidative stress and lead to activation of growth factors involved in inflammation [50,51]. There is no literature data which analyzed the influence of age on the level AGEs and sRAGE in the blood of hypertensive patients. However, Shapiro and co-authors evaluated the level of AGEs and *N*-(carboxymethyl)-lysine (CML) in LV and aorta of elderly canines with experimental hypertension (aged 8–11 years, old hypertensives) compared to young normal dogs (aged ≈ 1 year) [51]. The authors demonstrated a higher aortic CML in elderly dogs compared to young normal dogs. Furthermore, CML was essentially undetectable in young dogs. They also speculated that effect of CML and AGEs on LV properties may be more severe in the presence of coexisting diseases, such as diabetes or renal dysfunction [51]. Takahashi and co-authors estimated the effect of age and menopause on serum concentrations of pentosidine in young, and old subjects (140 healthy women aged 20–93 years) [52]. Pentosidine is one of the products formed through the Maillard reaction, the concentration of which increased during aging [52]. The authors found significantly higher values of serum pentosidine in women beyond the age of 50 compared to younger participants. Furthermore, the concentration of pentosidine was three times higher in women aged 80–93 years than in women aged 20–29 years. Although, we did not measure serum pentosidine, the age range of hypertensive patients and healthy volunteers is more limited. It is fact, that there is a significant difference in the average age between C and hypertensive patients but the lower age of healthy participants has a high probability of eliminating the effect of aging and various coexisting diseases on the oxidative modification of proteins. Based on our result, we may assume that the effect of age on key parameters is not significant in studied patients.

Finally, the integrative aim of the study, which combines the above-mentioned considerations, was to analyze which parameter may be an independent predictor of endothelium dysfunction evaluated by CEPCs and CECs numbers as well as CEPCs/CECs ratio. In a multivariate analysis, taking into consideration MH and RH patients, AGEs were associated with an increased CECs number and sRAGE was associated with and increased CEPCs/CECs ratio, independently of other factors considered, including hypertension type, SBP1, hsCRP, and renal function parameter. Based on our results, we conclude that both AGEs and sRAGE may be potential biomarkers of endothelial dysfunction in hypertensive patients. Interestingly, multivariate regression analysis revealed that AGEs may be an independent predictor of a condition and functioning of endothelium, whereas sRAGE may be classified as an independent biomarker of impaired/imbalanced endothelium homeostasis. Since both parameters are involved in endothelial dysfunction, we would expect that the AGEs/sRAGE ratio may play a similar role. However, our study revealed that the statistical significance of the AGEs/sRAGE ratio was lost after adjusting for the confounding factors we had proposed. It is likely that a larger number of participants would enhance the statistical significance of the AGEs/sRAGE ratio, yet further examination is essential.

## 4. Material and Methods

### 4.1. Patients

The study was conducted in a group of hypertensive patients (38 men and 22 women; mean age 55.57 ± 12.91) who had been admitted to the Department of Hypertension at the University of Medical Sciences in Poznan. The study protocol conforms to the ethical guidelines of the World Medical Association Declaration of Helsinki. This study was approved and conducted in accordance with the guidelines set by the Local Bioethical Committee of Poznan University of Medical Sciences (no. 163/17, 02 Febuary 2017). All patients qualified for this study underwent a detailed interview and a clinical examination. Written informed consent was obtained from all participants. Based on a pre-study interview, patients were divided into two groups: MH patients, 20 men and 10 women (mean age 52.87 ± 13.55), and RH patients, 19 men and 11 women (mean age 58.27 ± 11.85). Resistant arterial hypertension was diagnosed when the patients were unable to achieve target values of arterial blood pressure (< 140/90 mmHg), despite the use of at least 3 antihypertensive agents (including a diuretic) in maximum doses [3,4]. In abdominal ultrasound examinations from all MH and RH patients, computed tomography of the abdomen and Doppler ultrasound of the renal arteries were evaluated to exclude secondary causes of arterial hypertension. All study participants underwent transthoracic echocardiography. The control group (C) consisted of 23 subjects (8 women and 15 men), aged 25–51 (mean age: 32.80 ± 9.20), all of whom were normotensive blood donors in Regional Blood Centre in Poznan.

### 4.2. Blood Pressure Measurements

Clinical blood pressure (BP) was measured three times, at admission and at rest, in a supine position and in standard conditions, using an upper-arm blood pressure monitor (Omron 705IT).

### 4.3. Sample Collection

Blood samples were collected from MH and RH patients in the recumbent position after 10 min of rest. Two vacuum neutral tubes of blood were collected from each patient, one into Li-heparin (LH) anticoagulant tubes for ELISA analysis, and the other into ethylenediaminetetraacetic acid (EDTA) anticoagulant tubes for flow cytometric analysis. Blood was collected into LH tube first, and next into EDTA tube in order to minimize the number of endothelial cells phenotypically equivalent to CECs exfoliated during venipuncture procedures. Next, LH tubes were centrifuged at 3000 rpm for 15 min. Plasma samples were stored at the temperature of −80 °C until all of assays were performed. However, flow cytometric analysis was performed within 2 h of blood collection into EDTA tubes.

### 4.4. Biochemical Parameters

Hematological parameters such as: white blood cell count (WBC), red blood cell count (RBC), hemoglobin (HGB), blood platelets (PLT), neutrophils (NEUT), lymphocytes (LYMPH), and monocytes (MONO) were determined by Medonic M20 automatic analyzer (Clinical Diagnostic Solution, Inc., 1800 NW 65th Avenue Plantation, FL, USA). Blood biochemical analysis was performed using an EasyRA analyzer (Medica Corporation, 5 Oak Park Drive Bedford, MA, USA) and included the determination of creatinine (Cr), sodium (Na), glucose (G). Table 7 presents the demographic and biochemical characteristics of all patients that qualified for the study.

### 4.5. AGE Assay Kit (Cell Biolabs, Inc., San Diego, CA, USA)

In the first step of analysis, the plate was coated by an AGE conjugate. Next, AGE-BSA standards and the unknown samples were added to the ELISA plate, followed by incubation, the addition of an anti-AGE polyclonal antibody and finally the addition of an HRP conjugated secondary antibody. The absorbance was measured spectrophotometrically at 450 nm and the concentration of AGE adducts was calculated based on a predetermined AGE-BSA standard curve. The enzyme-linked-immunosorbent assay (ELISA) was carried out using Zenyth 200 Microplate Spectrophotometer (Anthos Labtec Instruments GmbH, Wals, Austria).

### 4.6. sRAGE (RayBiotech, Norcross, Peachtree Corners, GA, USA)

First, standards and unknown samples were pipetted into the walls coated by an antibody specific for human RAGE. After incubation and washing out, biotinylated antihuman RAGE antibody was added. Next, HRP-conjugated streptavidin, followed by 3,3,5,5′-tetramethylbenzidine (TMB) substrate was pipetted into each well. The addition of a stop solution changed the color from blue to yellow and the color intensity was measured spectrophotometrically at 450 nm. The enzyme-linked-immunosorbent assay kit was carried out using Zenyth 200 Microplate Spectrophotometer (Anthos Labtec Instruments GmbH, Wals, Austria).

### 4.7. Multicolor Flow Cytometry Analysis

Multicolor flow cytometry analysis was performed according to the method published by Szpera-Goździewicz et al. [53] with some modifications showed in previous study [23]. PE/Cy7 anti-human CD34 (BioLegend, London, United Kingdom), FITC anti-human CD146 (BioLegend), apc/Cy7 anti-human CD45 (BioLegend), and PE/Cy5 anti-human CD106 (BioLegend) mouse anti-human monoclonal antibodies were used for analysis. Two test tubes containing 100 of blood were prepared. To the one CD34, CD146, CD45, and CD133 were added, whereas another was used to determine the background of autofluorescence and non-specific antibody response. After incubation in dark, at room temperature, buffered lysing solution (FACS lysing solution, Becton Dickinson, San Jose, CA, USA) was added into both test tubes, followed by incubation for 10 min under the same conditions. Next, the lysis was stopped by addition of phosphate buffer, followed by centrifugation and decantation of supernatant. After elimination of the supernatant, the cell pellet was suspended in phosphate buffer. The evaluation of nucleated cells was obtained using a 6-color FACSCanto flow cytometer (BD Biosciences, San Jose, CA, USA), and data were analyzed using BD FACSDiva software. Each time before analysis, the flow cytometer was calibrated. The number of CECs was defined as positive for CD34, CD146 and negative for CD45 and CD133, with positive results for CD34, CD146, CD133 and negative results for CD45 as CEPCs.

### 4.8. Patient Exclusion Criteria

The exclusion criteria were as follows: secondary hypertension, white coat hypertension, myocardial infarction and revascularization within 6 months before the study, stroke and transient ischemic attack (TIA) within 6 months before the study, congestive heart failure with grade III-IV according to New York Heart Association grading (NYHA), chronic kidney disease (eGFR < 30 mL/min), addiction to alcohol and psychotropic substances, active cancer, or diabetes.

### 4.9. Statistical Analysis

The post-hoc analyses were carried out within this study project. Statistical analyses were conducted using GraphPad InStat or GraphPad Prism software 5.0 (Graph-Pad Software, San Diego, CA, USA). The normality of quantitative variables was tested using the Kolmogorov-Smirnow, Shapiro-Wilk or Pearson omnibus normality test. In the present study, the distribution of the data was analyzed using all three normality tests. But Pearson omnibus normality test has been given highest priority on the basis of recommendation of the producer. Normally distributed, continuous variables were analyzed using the Student’s t test. In contrast, One-way ANOVA analysis were used for the comparison of three studied groups. In this case, the Kruskal-Wallis test followed by Dunn’s Multiple Comparison Test or one-way analysis of variance was used, depending on data distribution. The strength of associations between different variables was tested using either the Pearson or Spearman correlation coefficients. In all cases, *p* value ≤ 0.05 was considered statistically significant. The relationship between AGEs, sRAGE or the AGEs/sRAGE ratio and endothelium dysfunction was evaluated using multivariate regression analyses. Five different models were proposed: before adjustment (model 1), after adjustment for type/grade of hypertension (model 2), after adjustment for SBP1 (model 3), after adjustment for hsCRP (model 4) and after adjustment for Cr (model 5), respectively.

## 5. Conclusions

The analysis of various parameters/factors confirmed that increased oxidative stress and inflammation are involved in vascular damage in pathogenesis of hypertension. Based on the present study, it may be concluded that both stages of hypertension contribute to the formation of advanced glycation end products. However, the lack of the gradation in AGEs according to the disease severity may suggest more intensified oxidative stress in MH patients despite lower complications than in RH patients. The decrease in sRAGE level and the elevation of the AGEs/sRAGE ratio in both groups may suggest that hypertensive patients are less protected against side effects of AGEs as a consequence of an insufficient competitive role of sRAGE against AGEs-RAGE axis. Finally, it may be concluded that the level of AGEs may be an independent predictor of the condition and functioning of endothelium, whereas sRAGE may be classified as a potential biomarker of inflammation and impaired/imbalanced endothelium homeostasis.

## Figures and Tables

**Table 1:** Comparison between patients categorized into quartiles according to AGEs, sRAGE, and the AGEs/sRAGE ratio.

Parameter	Quartile I	Quartile II and III	Quartile IV	
**MH**				
	AGEs/sRAGE < 0.2567	AGEs/sRAGE 0.2567–0.5501	AGEs/sRAGE > 0.5501	*p* value
CECs	198 (163–284)	126 (66–152)	74 (34–150) ^	0.0252 ^a^
CEPCs	413 (202–523)	140 (96–359)	83 (70–357) ^	0.0321 ^a^
**C**				
	AGEs < 38.28	AGEs 38.28–60.98	AGEs > 60.98	*p* value
sRAGE	185.50 ± 48.30	180.10 ± 47.20	126.30 ± 25.88 ^	0.0425 ^b^

^a^ Results shown as the median and interquartile range, Kruskull–Wallis test followed by Dunn’s multiple comparison test was used for comparison. ^b^ Results shown as mean ± standard deviation, one-way analysis of variance test was used for comparison. Significant differences: ^ Quartile I vs. Quartile IV; *p* ≤ 0.05.

**Table 2:** The correlations coefficients for AGEs, sRAGE, AGEs/sRAGE, CEPCs, CECs and CEPCs/CECs in the studied groups of patients.

**AGEs** *		
	*r*	Group of patients
sRAGE	−0.5169	C
sRAGE *		
	*r*	Group of patients
SBP1	−0.3735	MH
CECs	0.5591	MH
CEPCs	0.5703	MH
AGEs	−0.5169	C
	AGEs/sRAGE	
	*r*	Group of patients
CECs	−0.5983	MH
CEPCs	−0.6375	MH
	CECs	
	r	Group of patients
sRAGE	0.5591	MH
CEPCs	0.5997	MH
hsCRP	0.4598	RH
CEPCs	0.6715	RH
	CEPCs	
	r	Group of patients
CECs	0.5997	MH
sRAGE	0.5703	MH
CECs	0.5997	MH
CECs	0.6715	RH

*r*—correlation coefficient; * *p* ≤ 0.05.

**Table 3:** The influence of hsCRP concentration on AGEs, RAGEs, AGEs/RAGEs ratio, and SBP1 in MH and RH patients.

Group	hsCRP (mg/L)	AGEs (μg/mL)	sRAGE (pg/mL)	AGEs/RAGEs (μg/pg)	SBP1 (mmHg)	r
MH	<1 (*n* = 8)	54.39 ± 10.16	149.9 ± 75.1	0.325 (0.232–0.832)	141.8 ± 17.9	
	1 ≤ hsCRP < 3 (*n* = 12)	50.70 ± 7.08	169.30 ± 86.46	0.318 (0.199–0.494)	144.8 ± 16.5	−0.6197 (sRAGE) 0.6363 (AGEs/sRAGE)
	≥3 (*n* = 8)	53.55 ± 10.24	140.0 ± 67.7	0.414 (0.265–0.540)	141.9 ± 16.1	
RH	<1 (*n* = 5)	59.35 ± 8.00	184.3 ± 71.5	0.283 (0.258–0.586)	193.5 ± 21.8 *	
	1 ≤ hsCRP < 3 (*n* = 9)	48.64 ± 14.00	142.60 ± 64.43	0.418 (0.310–0.532)	178.3 ± 21.9 ^	
	≥3 (*n* = 11)	62.60 ± 14.89 ^a^	114.4 ± 33.2 ^b^	0.554 (0.390–0.852)	167.7 ± 21.0^#b^	

Data are presented as the mean ± standard deviation or median [range]. * *p* ≤ 0.05; hsCRP < 1 mg/L RH group vs. hsCRP < 1 mg/L MH group. ^ *p* ≤ 0.05; 1 mg/L ≤ hsCRP < 3 mg/L RH group vs. 1 mg/L ≤ hsCRP < 3 mg/L MH group. # *p* ≤ 0.05; hsCRP ≥ 3 mg/L RH group vs. ≥ 3 mg/L MH group. ^a^ *p* ≤ 0.05; 1 mg/L ≤ hsCRP < 3 mg/L RH group vs. hsCRP ≥ 3 mg/L RH group. ^b^ *p* ≤ 0.05; hsCRP < 1 mg/L RH group hsCRP ≥ 3 mg/L RH group. r—correlation coefficient.

**Table 4:** The influence gender on AGEs, RAGEs, AGEs/RAGEs ratio, hsCRP and SBP1 in MH and RH patients.

Group	Gender	Age (years)	AGEs (μg/mL)	sRAGE (pg/mL)	AGEs/RAGEs (μg/pg)	hsCRP (mg/L)	SBP1 (mmHg)
MH	Female (*n* = 10)	54.0 ± 13.5	52.88 ±6.97	160.90 ± 87.32	0.44 ± 0.27	2.7 (0.8–6.4)	149.10 ± 17.03
	Male (*n* = 20)	53.15 ± 14.18	50.36 ± 10.12	141.5 ± 59.2	0.46 ± 0.32	1.5 (0.9–3.53)	141.90 ± 15.53
RH	Female (*n* = 11)	58.82 ± 11.63	58.19 ± 6.13 ^	146.4 ± 53.9	0.44 ± 0.14	1.55 (0.73–4.9)	180.8 ± 24.3 ^
	Male (*n* = 19)	57.95 ± 12.28	57.26 ± 14.49 ^#^	165.3 ± 140.5	0.49 ± 0.29	6.1 (2.55−11.43) *^#^	168.5 ± 21.2 ^#^

Data are presented as the mean ± standard deviation or median (range). * *p* ≤ 0.05; the male RH group vs. the female RH group. ^ *p* ≤ 0.05; the female MH group vs. the female RH group. ^#^ *p* ≤ 0.05; the male MH group vs. the male RH group.

**Table 5:** The influence of age on AGEs, RAGEs, AGEs/RAGEs ratio, and SBP1 in MH and RH patients.

Group	Age	AGEs (μg/mL)	sRAGE (pg/mL)	AGEs/RAGEs (μg/pg)	hsCRP (mg/L)	SBP1 (mmHg)
MH	≤56 (*n* = 15)	50.61 ± 7.90	170.6 ± 74.2	0.29 (0.24–0.48)	1.5 (1.1–3.5)	140.7 ± 17.5
	> 56 (*n* = 15)	52.08 ± 10.30	126.6 ± 64.0	0.43 (0.27–0.84)	1.90 (0.85–5.65)	147.90 ± 15.05
RH	≤60 (*n* = 14)	58.01 ± 13.15	171.3 ± 151.6	0.43 (0.29–0.61)	5.05 (1.43–8.13)	176.6 ± 26.1
	> 60 (*n* = 16)	54.85 ± 14.25	152.9 ± 69.3	0.46 (0.26–0.58)	2.2 (1.2–4.9)	172.4 ± 19.5

Data are presented as the mean ± standard deviation or median (range).

**Table 6:** Multivariate analysis of relation between endothelium dysfunction and AGEs, sRAGE, and AGEs/sRAGE ratio.

Model	CEPCs		CECs		CEPCs/CECs	
	Coefficient	*p*	Coefficient	*p*	Coefficient	*p*
**Model 1**						
AGEs	−5.679	0.4110	−1.638	0.3159	−0.0300	0.4875
sRAGE	0.03253	0.9747	0.2171	0.3723	−0.0025	0.7009
AGEs/sRAGE	−276.26	0.5130	−0.6247	0.9950	−0.6646	0.8017
**Model 2**						
AGEs	−5.744	0.4197	−1.688	0.3158	−0.03187	0.4753
sRAGE	0.03323	0.9744	0.2176	0.3755	−0.00245	0.7056
AGEs/sRAGE	−274.61	0.5209	0.6357	0.9950	−0.6197	0.8171
**Model 3**						
AGEs	−7.069	0.3037	−1.573	0.3432	−0.03957	0.3564
sRAGE	−0.1109	0.9130	0.2239	0.3636	−0.003458	0.5864
AGEs/sRAGE	−337.00	0.4204	2.233	0.9823	−1.080	0.6789
**Model 4**						
AGEs	−11.355	0.1902	−4.027	0.0451	0.006681	0.8919
sRAGE	0.6907	0.7381	0.5898	0.2158	−0.004173	0.7243
AGEs/sRAGE	−18.047	0.9751	96.908	0.4657	−0.9481	0.7749
**Model 5**						
AGEs	−4.773	0.4956	−1.757	0.2917	−0.007762	0.8444
sRAGE	−0.4750	0.6913	0.2836	0.3193	−0.01498	0.0302
AGEs/sRAGE	−408.23	0.3674	16.674	0.8762	−3.915	0.1291

**Table 7:** Demographic characteristics and biochemical parameters in the plasma of MH, RH and C groups.

Parameters	MH	RH	C	*p* Value
Age (years)	52.87 ± 13.55 *	58.27 ± 11.85 **	32.80 ± 9.20	0.001 ^a^
Gender F/M (n)	10/20	12/18	8/15	NS ^b^
BMI (kg/m^2^)	28.53 ± 5.14	30.09± 5.73	26.90 ± 4.80	NS ^a^
SBP1 [mm Hg]	144.5 ± 16.5 *	173.0 ± 23.0 **	121.4 ± 2.7	<0.0001 ^a^
Red blood cells (RBC) [10^12^/L]	4.82 (4.46–5.13) *	4.60 (4.34–5.07) **	5.01 (4.89–5.33)	0.026 ^c^
White blood cells (WBC) [10^9^/L]	7.22 (5.68–8.95) *	6.95 (5.69–8.60) **	6.00 (5.20–7.05)	0.009 ^c^
Platelets (PLT) [10^9^/L]	226.0 ± 56.2	221.0 ± 51.8	236.0 ± 54.0	NS ^a^
Neutrophils (NEUT) [10^9^/L]	4.48 (3.41–5.72) *	4.42 (3.55–5.85) **	3.16 (2.52–3.53)	<0.0001 ^c^
Lymphocytes (LYMPH) [10^9^/L]	1.63 (1.36–2.36)	1.82 (1.37–2.15)	2.16 (1.77–2.50)	NS ^c^
Monocytes (MONO) [10^9^/L]	0.47 (0.34–0.53) *	0.45 (0.28–0.67)	0.56 (0.43–0.63)	0.036 ^c^
Hemoglobin (HGB) [mmol/L]	9.04 ± 0.86 *	8.84 ± 0.86 **	14.90 ± 1.26	<0.0001 ^a^
Creatinine (Cr) [μmol/L]	85.0 (66.0–92.4)	83.45 (70.58–112.10)	70.20 (62.1–94.90)	NS ^c^
Glucose (G) [mmol/L]	5.59 (5.03–6.30)	5.58 (5.20–6.38)	4.98 (4.12–5.10)	NS ^c^
hsCRP [mg/L]	1.75 (0.95–3.58) *	4.0 (1.47–8.03) **	1.0 (0.80–1.20)	0.0001 ^c^
CECs	126 (67–198) *	113 (64–233) **	50 (17–78)	<0.0001 ^c^
CEPCs	167(106–411)	164 (101−320)	153 (102–232)	NS ^c^
CEPCs/CECs ratio	1.60 (1.01–2.25) *	1.35 (1.09–1.98) **	3.25 (2.03–14.11)	0.012 ^c^

F—female, M—male, BMI—body mass index, SBP1—office systolic blood pressure-admission, hsCRP—high-sensitive C-reactive protein; **^a^** Results shown as mean ± standard deviation, one-way ANOVA test (one-way analysis of variances) was used for comparison; ^b^ Categorical data, Fischer’s exact test was used for comparison; ^c^ Results shown as median and interquartile range, one-way ANOVA test (Kruskal-Wallis) test was used for comparison; NS—not statistically significant; * MH vs. C *p* ≤ 0.05; ** RH vs. C *p* ≤ 0.05.
